# Fluoroquinolone Resistance in *Salmonella*: Deciphering Molecular Mechanisms, Global Dissemination Dynamics, and Clinical Countermeasures

**DOI:** 10.3390/microorganisms14102198

**Published:** 2026-10-01

**Authors:** Meiqi Chen, Shuojie Lv, Wanying Zheng, Qingjing Wang

**Affiliations:** Key Laboratory of Artificial Organs and Computational Medicine of Zhejiang Province, Key Laboratory of Pollution Exposure and Health Intervention of Zhejiang Province, Shulan International Medical College, Zhejiang Shuren University, Hangzhou 310015, China

**Keywords:** fluoroquinolones, *Salmonella*, resistance mechanisms, chromosomal mutations, dissemination mechanisms, plasmid-mediated quinolone resistance

## Abstract

Increasing clinical use of fluoroquinolone antibiotics has led to a rapid surge in fluoroquinolone-resistant *Salmonella* strains, markedly constraining the therapeutic armamentarium and increasing treatment failure risks. Epidemiological surveillance reveals high prevalence rates of resistant isolates across Asia, Africa, Europe, and the Americas, with infection-associated mortality remaining persistently elevated—a situation that poses a formidable threat to global public health. Despite this urgency, systematic investigations elucidating the precise molecular underpinnings of resistance and the mechanistic pathways governing transnational dissemination remain insufficient. This comprehensive review delineates the resistance and propagation mechanisms of fluoroquinolone-resistant *Salmonella*, with particular emphasis on the synergistic interplay between chromosomal mutations in *gyrA*/*parC* and the overexpression of efflux pump systems, alongside the horizontal transfer of plasmid-mediated quinolone resistance (PMQR) determinants—including *qnr*, *aac(6′)-Ib-cr*, and *oqxAB*—facilitated by integrons, transposons, and insertion sequences (ISs). The intricate complexity of these resistance mechanisms and dissemination routes poses substantial challenges to clinical therapeutics, rendering conventional antibiotic regimens progressively ineffective. Accordingly, this review further explores the development of novel therapeutic modalities and antimicrobial agents, aiming to inform evidence-based prevention and control strategies.

## 1. Introduction

*Salmonella* constitutes a preeminent foodborne Gram-negative bacterial pathogen of significant public health concern. To date, more than 2500 serovars have been identified, broadly taxonomized into typhoidal *Salmonella* (e.g., S. Typhi) and nontyphoidal *Salmonella* (NTS) on the basis of antigenic architecture and clinical manifestations [1]. Typhoidal *Salmonella* accounts for approximately 11 million cases and 110,000 fatalities annually worldwide. Clinically, typhoid fever is characterized by sustained bacteremia and a constellation of constitutional symptoms that frequently defy differentiation from other febrile illnesses [2]. NTS infections manifest diversely as gastroenteritis (food poisoning), septicemia, and localized suppurative infections, with enterocolitis representing the predominant clinical presentation, causing an estimated 10 million cases of gastroenteritis and 116,800 deaths per year globally [3,4,5,6].

The therapeutic management of fluoroquinolone-resistant *Salmonella* infections represents a focal challenge in contemporary antimicrobial resistance (AMR) research. According to the most recent World Health Organization (WHO) priority pathogen list, both fluoroquinolone-resistant typhoidal *Salmonella* and fluoroquinolone-resistant nontyphoidal *Salmonella* have been classified as high-priority pathogens, underscoring their severe public health implications and the exigent demand for novel antimicrobial agents.

Antimicrobial resistance in *Salmonella*, however, extends well beyond fluoroquinolones, encompassing virtually all major antibiotic classes in clinical use, including β-lactams, aminoglycosides, tetracyclines, and sulfonamides, with multidrug-resistant and extensively drug-resistant isolates increasingly reported worldwide. Among these classes, fluoroquinolones occupy a particularly prominent position and are among the most widely prescribed antibiotics globally. Based on their antimicrobial spectrum and developmental chronology, fluoroquinolones are conventionally classified into four generations: the non-fluorinated first-generation agent nalidixic acid; the second-generation early fluoroquinolones, including ciprofloxacin, norfloxacin, and ofloxacin, which are highly active against Gram-negative bacilli; the third-generation agent levofloxacin, which offers improved activity against Gram-positive pathogens; and the fourth-generation agents, exemplified by moxifloxacin, which feature expanded activity against anaerobes and atypical pathogens. Owing to their broad spectrum, favorable oral bioavailability, and bactericidal activity, fluoroquinolones have become the first-line empirical therapy for both enteric and invasive salmonellosis, which in turn has driven intense selective pressure and the rapid emergence of resistance.

The global distribution of resistant *Salmonella* is notably heterogeneous, with disproportionately high disease burdens observed in low- and middle-income countries (LMICs) [7]. The emergence of multidrug-resistant (MDR) typhoid fever has prompted the widespread adoption of fluoroquinolones as first-line empirical therapy; however, fluoroquinolone nonsusceptibility rapidly emerged and became endemic, particularly in South and Southeast Asia. High-level ciprofloxacin resistance (CipR) in S. Typhi was concentrated in South Asia but was not uniform across countries: in a meta-analysis of approximately 13,000 genomes, CipR was common (≥10%) in India, Nepal, and Pakistan, whereas it remained emerging (3–6%) in Bangladesh; in India, annual CipR prevalence was estimated at 19–27% from 2014 onward [8]. This should be distinguished from ciprofloxacin non-susceptibility (CipNS), which exceeded 95% in India and Bangladesh, stabilized at 85–95% in Nepal after 2011, and exceeded 95% in Pakistan after 2012 [8]. Separately, a single-center retrospective study in Kolkata (June 2017–June 2022) reported phenotypic fluoroquinolone resistance in all *Salmonella* Typhi and *Salmonella* Paratyphi A isolates from 2019 onward; given the small annual isolate numbers, this finding should not be extrapolated to national prevalence [9]. Furthermore, the dissemination of fluoroquinolone-resistant *Salmonella* transcends local boundaries, demonstrating transboundary and intercontinental propagation patterns. Although the prevalence of fluoroquinolone-resistant strains remains relatively modest in Africa, incremental reports from Kenya, Tanzania, and Malawi have documented the intercontinental spread of the H58 haplotype from Asia to Africa—a clone harboring resistance-associated mutations that has substantially exacerbated the regional resistance burden [10]. Consequently, addressing this challenge is imperative for improving public health outcomes in affected regions. The WHO prioritization further emphasizes the necessity of comprehensive, multisectoral public health interventions encompassing universal access to high-quality, affordable infection prevention, rapid diagnostics, and appropriate therapeutic strategies, thereby reaffirming the critical importance of preserving fluoroquinolone efficacy in *Salmonella* infection management [11].

From a biochemical perspective, fluoroquinolone resistance in *Salmonella* arises from the convergence of several distinct yet interacting molecular mechanisms. The principal target-based mechanism involves stepwise chromosomal mutations—most frequently single-nucleotide polymorphisms within the quinolone resistance-determining regions (QRDRs) of *gyrA* and parC, which encode the DNA gyrase and topoisomerase IV subunits, respectively—thereby reducing drug binding affinity; these mutations are frequently accompanied by the overexpression of multidrug efflux pump systems, such as AcrAB-TolC, that lower intracellular fluoroquinolone concentrations. In parallel, plasmid-mediated quinolone resistance (PMQR) determinants, including the *qnr* target-protection proteins, the *aac(6′)-Ib-cr* fluoroquinolone-modifying acetyltransferase, and the *oqxAB* efflux pump genes, confer low-level resistance and promote the selection of higher-level chromosomal resistance. These PMQR elements are disseminated horizontally among *Salmonella* serovars and across bacterial species via mobile genetic elements—namely integrons, transposons, and insertion sequences (ISs)—that capture, excise, and reintegrate resistance gene cassettes. Collectively, target modification, enzymatic inactivation, active efflux, and mobile-element-driven horizontal gene transfer constitute the biochemical machinery underlying the emergence, accumulation, and transnational spread of fluoroquinolone-resistant *Salmonella*.

Since the most recent comparable syntheses—genomic analyses of fluoroquinolone-resistant *Salmonella* and reviews centered largely on chromosomal QRDR mechanisms—several developments warrant an updated, mechanism-integrated review: (i) the emergence and intercontinental expansion of high-level ciprofloxacin-resistant and extensively drug-resistant S. Typhi clones carrying PMQR determinants such as qnrS alongside QRDR mutations; (ii) the shift from phenotype-based definitions of ciprofloxacin non-susceptibility to genome-informed classifications distinguishing CipNS from high-level CipR; and (iii) the rapid maturation of CRISPR-based diagnostics, phage-based delivery systems, and efflux-pump-targeted adjuvants as candidate countermeasures. Accordingly, the objective of this review is to provide a comprehensive and integrative overview of the biochemical and molecular basis of fluoroquinolone resistance in *Salmonella*. Specifically, we summarize the contributions of chromosomal QRDR mutations and efflux pump overexpression to resistance phenotypes, delineate the biochemical functions of PMQR determinants and their dissemination through integrons, transposons, and insertion sequences, and examine the transnational propagation routes of resistant strains. On this basis, we further discuss emerging therapeutic strategies and novel antimicrobial agents directed against these biochemical pathways, with the aim of informing evidence-based prevention and control measures against fluoroquinolone-resistant *Salmonella*.

## 2. Resistance Mechanisms

The increasing global utilization of broad-spectrum antibiotics has intensified the severity of fluoroquinolone resistance in *Salmonella*, with resistance mechanisms becoming increasingly multifactorial. These mechanisms include chromosomally mediated target-site mutations, overexpression of multidrug efflux pumps, enzymatic drug modification, reduced outer membrane permeability, and plasmid-mediated quinolone resistance (PMQR).

### 2.1. Chromosomally Mediated Mutations

Chromosomal mutations represent the principal mechanism conferring high-level fluoroquinolone resistance in *Salmonella*, primarily involving mutations within the quinolone resistance-determining regions (QRDRs) of DNA topoisomerase IV (*parC*, *parE*) and DNA gyrase (*gyrA*, *gyrB*) [7,11,12,13,14]. These mutations decrease the binding affinity of fluoroquinolones for bacterial topoisomerases and DNA gyrase, thereby attenuating antimicrobial efficacy.

DNA topoisomerase IV, a heterotetrameric enzyme composed of two ParC and two ParE subunits [(ParC-ParE)_2_], constitutes a primary target for fluoroquinolone action. Mutations within *parC* and *parE* alter the three-dimensional conformation of topoisomerase IV, modifying drug-binding sites and consequently diminishing antibiotic-binding capacity [15,16]. Molecular amplification and sequencing analyses revealed that mutations most frequently occur at amino acid position 80 of the *parC* gene, where serine is substituted by arginine (Ser80Arg), with less frequent substitutions observed at Glu84 or Ser83 [17,18,19]. Additionally, the Ser458Pro mutation in parE appears to be relatively prevalent. Isolates harboring both Ser80Arg and Ser458Pro mutations presented significantly elevated minimum inhibitory concentrations (MICs) against ciprofloxacin and related fluoroquinolones [19].

DNA gyrase, a tetrameric complex comprising two GyrA and two GyrB subunits [(GyrA-GyrB)_2_], also serves as a critical fluoroquinolone target. Fluoroquinolones typically exert bactericidal activity by binding to the gyrase–DNA complex and inhibiting strand cleavage and religation reactions. Mutations in both *gyrA* and *gyrB* are strongly associated with resistance phenotypes [20,21]. A systematic review and meta-analysis investigating 6103 *Salmonella* isolates from Africa reported an aggregated *gyrA* mutation prevalence of 5.7%, predominantly involving substitutions at two critical amino acid positions: Ser83Phe/Leu and Asp87Gly/Asn [22]. Although mutations at GyrB positions Ser464Tyr and Ser464Phe occur at lower frequencies than GyrA mutations do, they nonetheless contribute incrementally to resistance by compromising gyrase functionality and reducing the inhibitory effects of fluoroquinolones on bacterial DNA replication [23,24,25,26].

In addition to single-site mutations, synergistic epistatic interactions between mutations at distinct loci result in combined resistance effects that exceed simple additive contributions. In Asian typhoidal *Salmonella*, QRDR mutations in *gyrA* and *parC* are frequently observed and significantly correlate with fluoroquinolone resistance. In S. Typhi isolates harboring the Ser83Phe-Asp87Asn double mutation (together with additional *parC* mutations), three distinct single mutations at codon 87 of *gyrA* demonstrate selective advantages when combined with either the Ser83Phe mutation or the Ser80Ile *parC* mutation. Notably, the Ser83Phe and Asp87Asn combination had the most pronounced synergistic effect, with the resulting phenotypic interaction enhancing bacterial fitness by 6.6% [26], thereby facilitating the propagation and maintenance of resistance. Studies of ciprofloxacin-resistant S. Typhi and *Salmonella* Paratyphi A isolates from India have demonstrated that single *gyrA* mutations (Ser83Phe or Ser83Tyr) are associated with reduced ciprofloxacin susceptibility (MICs of 0.125–1 mg/L), whereas additional *parC* mutations (Ser80Ile, Ser80Arg, Asp69Glu, or Gly78Asp) correlate with further increases in the ciprofloxacin MIC (≥0.5 mg/L) [27]. Investigations in Japan involving 12 human-derived and 5 nonhuman-derived highly fluoroquinolone-resistant *Salmonella* Typhimurium isolates revealed that nearly all strains presented identical mutation profiles, including Ser83Phe and Asp87Asn in *gyrA* and Ser80Ile in *parC*. Strains initially harboring a single Ser83Phe mutation in *gyrA* subsequently acquired additional mutations at Asp87Asn (*gyrA*) and Ser80Ile (*parC*), culminating in high-level resistance [18,28]. Among Thai patients with systemic salmonellosis, third-generation cephalosporin- and/or fluoroquinolone-resistant *Salmonella* Typhimurium isolates harbored mutations in *gyrA* (Asp87Tyr and Asp87Gly) and *parC* (Thr57Ser) [29]. In African studies, *gyrA* mutations at codon 83 (Ser83Phe/Tyr) and codon 87 (Asp87Gly/Asn) predominated [30], whereas *parC* mutations occurred primarily at codon 80 (Ser80Ile/Arg), albeit at lower detection frequencies; nevertheless, *parC* mutations frequently cooccur with *gyrA* mutations to potentiate resistance [31]. The cumulative acquisition of multiple amino acid substitutions increases resistance to high-level phenotypes [23,24].

These mutations may exist individually or in combination [13], altering drug-binding sites and cooperatively elevating MICs to fluoroquinolones, thereby stabilizing the expression of resistant phenotypes and exacerbating the overall resistance burden in *Salmonella*.

### 2.2. Efflux Pump Overexpression and Enzymatic Modification

In addition to target-site alterations, increased expression of efflux-pump systems plays a critical role in fluoroquinolone resistance. Studies examining *Salmonella* Enteritidis have revealed an association between *crp* gene deletion and fluoroquinolone resistance [32,33]. The *crp* gene encodes the cyclic AMP (cAMP) receptor protein, a global transcriptional regulator that indirectly modulates bacterial drug susceptibility by influencing the expression of multiple genes, including those encoding outer membrane porins [34,35]. In crp-deficient mutants, decreased expression of the outer membrane porins OmpA and OmpF was observed concomitantly with upregulated expression of the efflux pump gene acrB; these combined alterations increase fluoroquinolone extrusion [36], reduce intracellular drug concentrations, and consequently increase resistance levels [33]. Furthermore, Western blot analyses have demonstrated elevated AcrA protein expression in resistant strains, indicating that overexpression of the AcrAB-TolC efflux system substantially contributes to the resistance phenotype [11,16]. AcrAB-like efflux pump overexpression is directly associated with increased fluoroquinolone MICs. Experimental application of the efflux pump inhibitor Phe-Arg-β-naphthylamide (PAβN) significantly reduced ciprofloxacin MICs in resistant strains, confirming the functional involvement of efflux systems in fluoroquinolone resistance [21,25].

**Figure 1:**
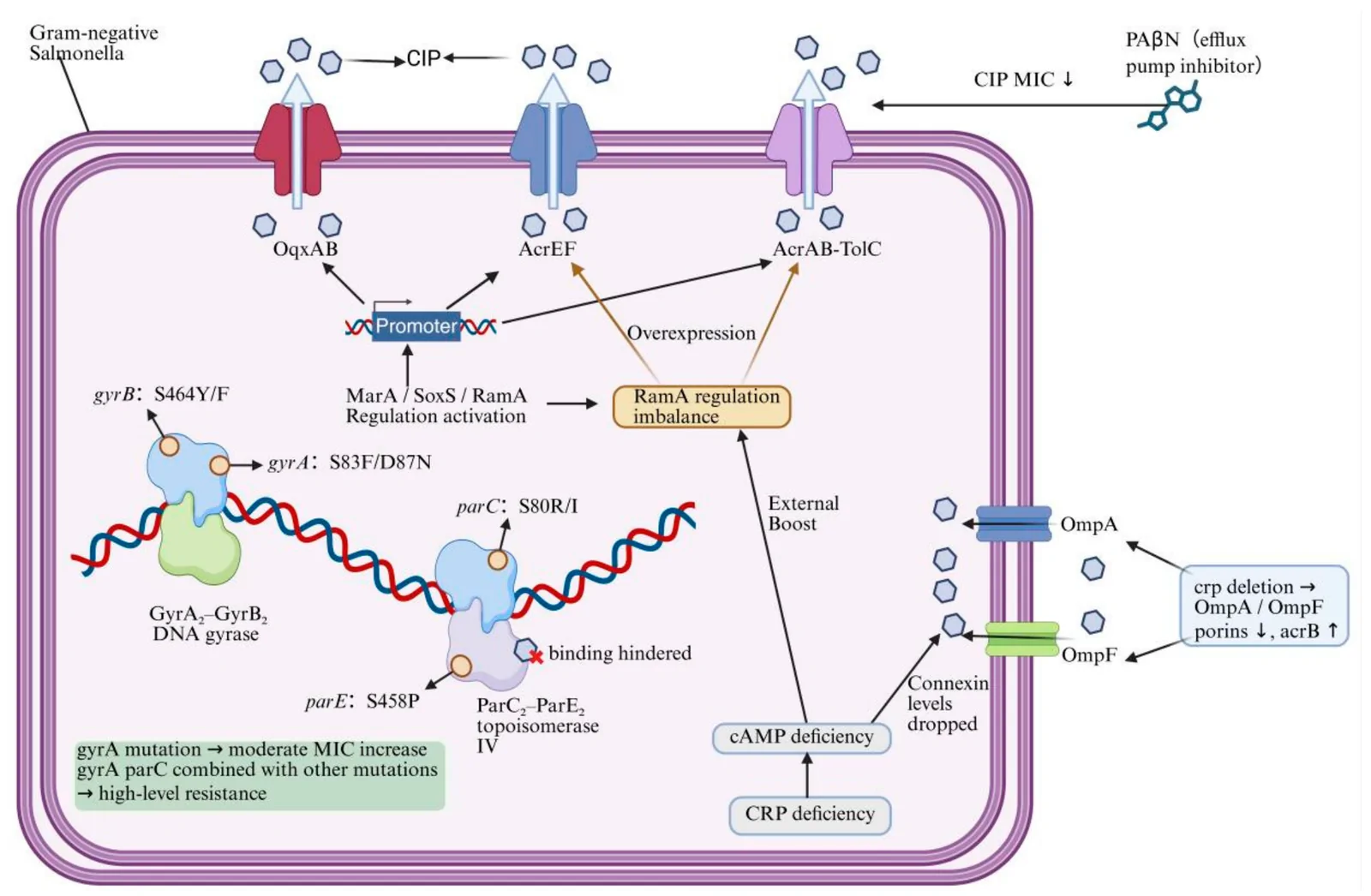
Schematic illustration of chromosomal fluoroquinolone resistance mechanisms in *Salmonella*. Mutations in the quinolone resistance-determining regions (QRDRs) of DNA gyrase (gyrA: Ser83Phe/Asp87Asn; gyrB: Ser464Tyr/F) and topoisomerase IV (parC: Ser80Arg/I; parE: Ser458Pro) alter the three-dimensional conformation of target enzymes. This sterically hinders fluoroquinolones (e.g., ciprofloxacin, CIP) from binding to the enzyme–DNA complex, thereby reducing drug efficacy. Synergistic interactions between gyrA and parC mutations significantly increase the minimum inhibitory concentration (MIC). Additionally, deletion of the crp gene leads to cAMP deficiency, which downregulates the expression of outer membrane porins (OmpA/OmpF), reducing drug influx. Concurrently, this regulatory imbalance upregulates efflux pump genes, further contributing to resistance. Furthermore, transcriptional regulators (MarA, SoxS, RamA) activate promoters to drive the overexpression of RND superfamily efflux pumps (OqxAB, AcrEF, and AcrAB-TolC). These pumps actively extrude intracellular drugs via proton gradients. The use of efflux pump inhibitors (e.g., PAβN) can restore susceptibility by blocking this extrusion. Abbreviations: CIP: ciprofloxacin; GyrA/GyrB: DNA gyrase subunit A/B; ParC/ParE: topoisomerase IV subunit C/E; MIC: minimum inhibitory concentration; PAβN: Phe-Arg-β-naphthylamide; CRP: cAMP receptor protein.Note: →, lead to; ↑, increase/upregulation; ↓, decrease/downregulation. CRP deficiency leads to less CIP entering through OmpA/OmpF porins and more acrB efflux.

The expression of efflux pumps is regulated by transcriptional activators such as MarA, SoxS, and RamA, which bind to promoter regions of efflux pump operons—including *oqxAB*—thereby activating transcription and increasing OqxA and OqxB protein expression, which enhances pump functionality. Mutant RamA expression is directly associated with the overexpression of the multidrug efflux pumps AcrAB and AcrEF and can confer multidrug resistance phenotypes [37] (Figure 1).

### 2.3. Plasmid-Mediated Quinolone Resistance (PMQR)

**Figure 2:**
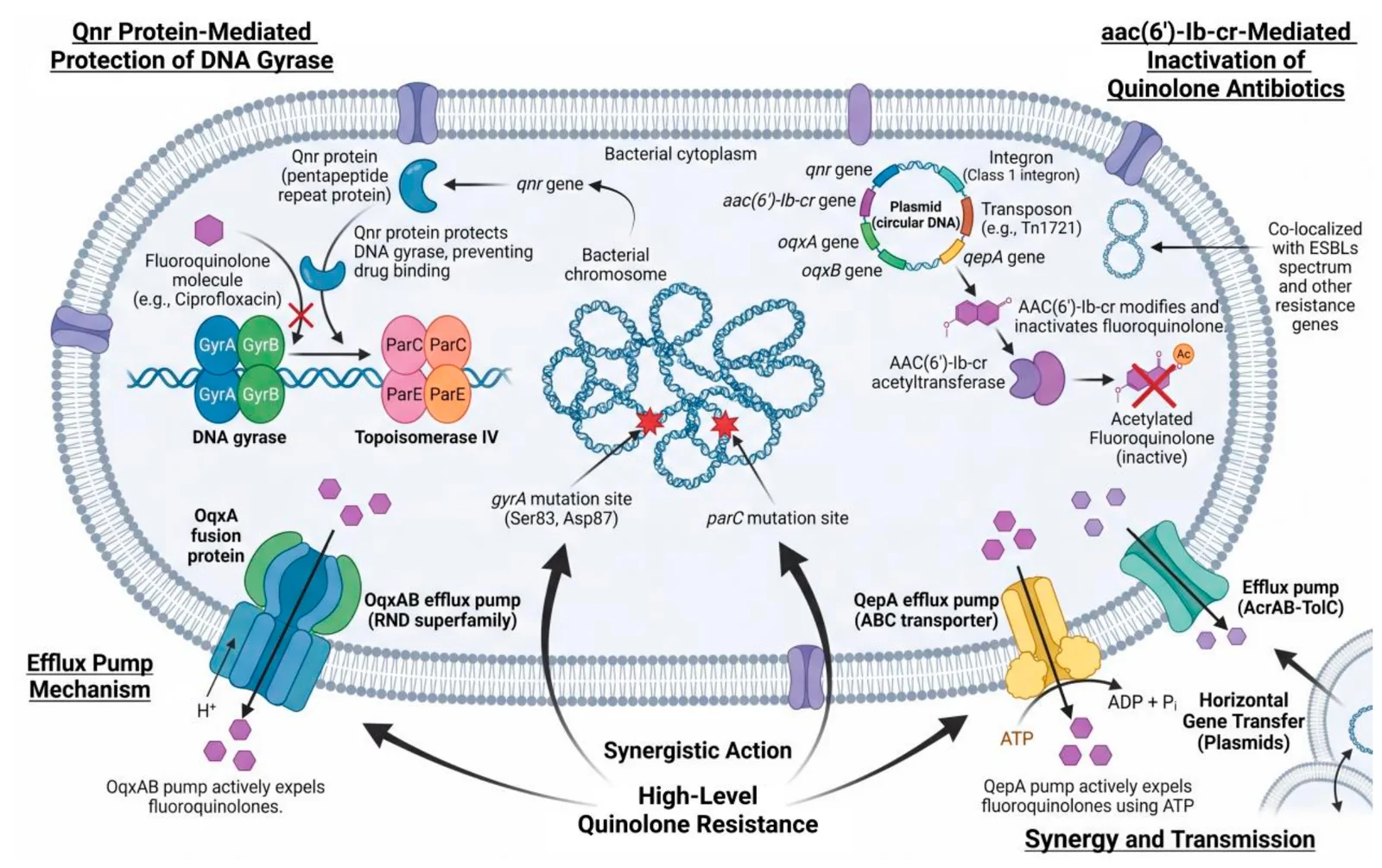
Schematic illustration of plasmid-mediated quinolone resistance (PMQR) mechanisms and their synergism with chromosomal mutations in *Salmonella*. The Qnr pentapeptide repeat protein (PPR) binds to DNA gyrase (GyrA/GyrB) and topoisomerase IV (ParC/ParE), sterically hindering fluoroquinolones (e.g., ciprofloxacin) from forming a ternary complex with target enzymes and DNA. Additionally, the AAC(6′)-Ib-cr acetyltransferase recognizes and acetylates piperazine-containing quinolones, leading to drug inactivation. Furthermore, the RND superfamily efflux pump OqxAB, ABC transporter QepA, and AcrAB-TolC system extrude intracellular drugs via a proton gradient and ATP hydrolysis, respectively. Chromosomal quinolone resistance-determining region (QRDR) mutations in *gyrA* (Ser83, Asp87) and parC synergize with PMQR genes, markedly increasing the minimum inhibitory concentration (MIC) and driving high-level fluoroquinolone resistance and horizontal transmission. DNA: deoxyribonucleic acid, GyrA/GyrB: DNA gyrase subunit A/B, ParC/ParE: topoisomerase IV subunit C/E, RND: resistance-nodulation-division, ABC: ATP-binding cassette, AAC(6′)-Ib-cr: aminoglycoside acetyltransferase 6′-N-acetyltransferase type Ib-cr variant. Note: ×, Lose activity; Fluoroquinolone drugs lose their activity after being acetylated.

Plasmid-mediated quinolone resistance (PMQR) operates through three primary mechanisms: Qnr protein-mediated protection of DNA gyrase, AAC(6′)-Ib-cr-mediated enzymatic inactivation of fluoroquinolones, and OqxAB/QepA efflux pump-mediated drug extrusion [7,38,39,40]. These resistance determinants typically colocalize with chromosomal QRDR mutations (e.g., in *gyrA* and *parC*) to produce high-level fluoroquinolone resistance and can disseminate horizontally among strains via conjugative plasmids, thereby increasing resistance transmission risk [41] (Figure 2).

#### 2.3.1. Qnr Protein-Mediated Gyrase Protection

Qnr proteins constitute a family of pentapeptide repeat proteins (PRPs) that shield DNA gyrase from fluoroquinolone inhibition. On the basis of amino acid sequence homology [42,43], the Qnr family is classified into distinct groups, including QnrA, QnrB, QnrC, QnrD, and QnrS [39,44]. Typically, Qnr proteins (approximately 200 amino acids) are composed of AXXXG pentapeptide repeats that fold into a right-handed β-helix structure. Through specific binding to the GyrA/GyrB subunits of DNA gyrase [32], Qnr proteins obstruct the formation of the quinolone–gyrase–DNA ternary complex; point mutations within this region can substantially alter drug–target interactions, thereby preventing fluoroquinolone binding and reducing susceptibility [42]. X-ray crystallographic studies have demonstrated that the third helical repeat unit of Qnr interacts with the QRDR of GyrA (specifically at key residues Ser83 and Asp87), directly interfering with the drug-binding pocket [45,46]. Although Qnr-mediated resistance typically increases fluoroquinolone MICs by only 2- to 8-fold—generally insufficient to breach clinical resistance thresholds—it synergizes with QRDR mutations (e.g., Ser83Phe, Asp87Gly) and AcrAB-TolC efflux pump overexpression to accelerate the acquisition of high-level resistance [40,45,47]. Epidemiological data from China indicate that *qnrS1* is detected in 19.25% of chicken-derived *Salmonella* isolates, with *qnrB6* present at 1.89%; these genes frequently colocalize with extended-spectrum β-lactamases (ESBLs) and *aac(6′)-Ib-cr* on IncHI2-type plasmids, suggesting potential transmission risks associated with multidrug resistance gene complexes [48].

#### 2.3.2. AAC(6′)-Ib-cr-Mediated Fluoroquinolone Inactivation

The *aac(6′)-Ib-cr* gene encodes a bifunctional aminoglycoside 6′-N-acetyltransferase variant derived from the wild-type AAC(6′)-Ib enzyme [39]. The introduction of two critical amino acid substitutions within the active site enables AAC(6′)-Ib-cr to recognize and acetylate fluoroquinolones bearing a piperazine substituent—notably ciprofloxacin and ofloxacin—resulting in drug inactivation and conferring low-level resistance [49]. Genetically, *aac(6′)-Ib-cr* is typically located on plasmids harboring class 1 integrons or Tn1721 transposons and frequently colocalizes with β-lactamase genes (e.g., *bla*CTX-M) and *qnr* determinants on IncFIA, ColE1-like (pMdT1), or IncN-type plasmids [50], disseminating horizontally among bacteria through transformation, transduction, and conjugation [44,51]. Surveillance studies in China have revealed that 37.1% of *Salmonella* Typhimurium isolates from hospitalized pediatric diarrhea patients carry *aac(6′)-Ib-cr*, with the acquisition of this gene increasing ciprofloxacin MICs by approximately 4-fold [52]. Under sustained fluoroquinolone selective pressure, bacterial populations harboring *aac(6′)-Ib-cr* exhibit progressively elevated resistance levels.

#### 2.3.3. OqxAB and QepA Efflux Pumps

The oqxAB and qepA genes encode proteins belonging to multidrug efflux pump systems. OqxAB, encoded by oqxA and oqxB, belongs to the resistance-nodulation-division (RND) superfamily of transporter complexes and utilizes the proton motive force to drive drug extrusion [53,54], increasing ciprofloxacin MICs by 10- to 16-fold. The oqxAB gene cluster is frequently mobilized by IS26 insertion elements into chromosomal loci or IncF/IncHI2-type plasmids and commonly colocalizes with β-lactamase and *aac(6′)-Ib-cr* resistance genes, substantially increasing the transmission risk of multidrug resistance [55,56]. In clinical *Salmonella* Typhimurium isolates recovered in Hong Kong, OqxAB and AAC(6′)-Ib-cr were shown to mediate the development of ciprofloxacin resistance (CIP MIC ≥ 1 μg/mL), increasing bacterial survival under high-dose ciprofloxacin exposure [57]. The *qepA* gene encodes a multidrug efflux pump whose expression is induced by diverse environmental factors, including antibiotic pressure and oxidative stress [58]. The mechanism of resistance to QepA is primarily mediated through conformational alterations that facilitate drug extrusion, thereby reducing the concentration of intracellular fluoroquinolone [59]. The *qepA* gene frequently colocalizes with class I integrons, increasing its dissemination potential among clinical and environmental isolates [60,61].

## 3. Dissemination Mechanisms

The rapid global diffusion of fluoroquinolone-resistant *Salmonella* is driven by a complex interplay of plasmids, integrons, genomic islands, transposons, insertion sequences (ISs), and antibiotic selection pressures, which collectively facilitate efficient horizontal gene transfer (HGT) of resistance determinants [62].

### 3.1. Plasmid-Mediated Horizontal Transfer

#### 3.1.1. Plasmid Conjugation and Mobilization

**Figure 3:**
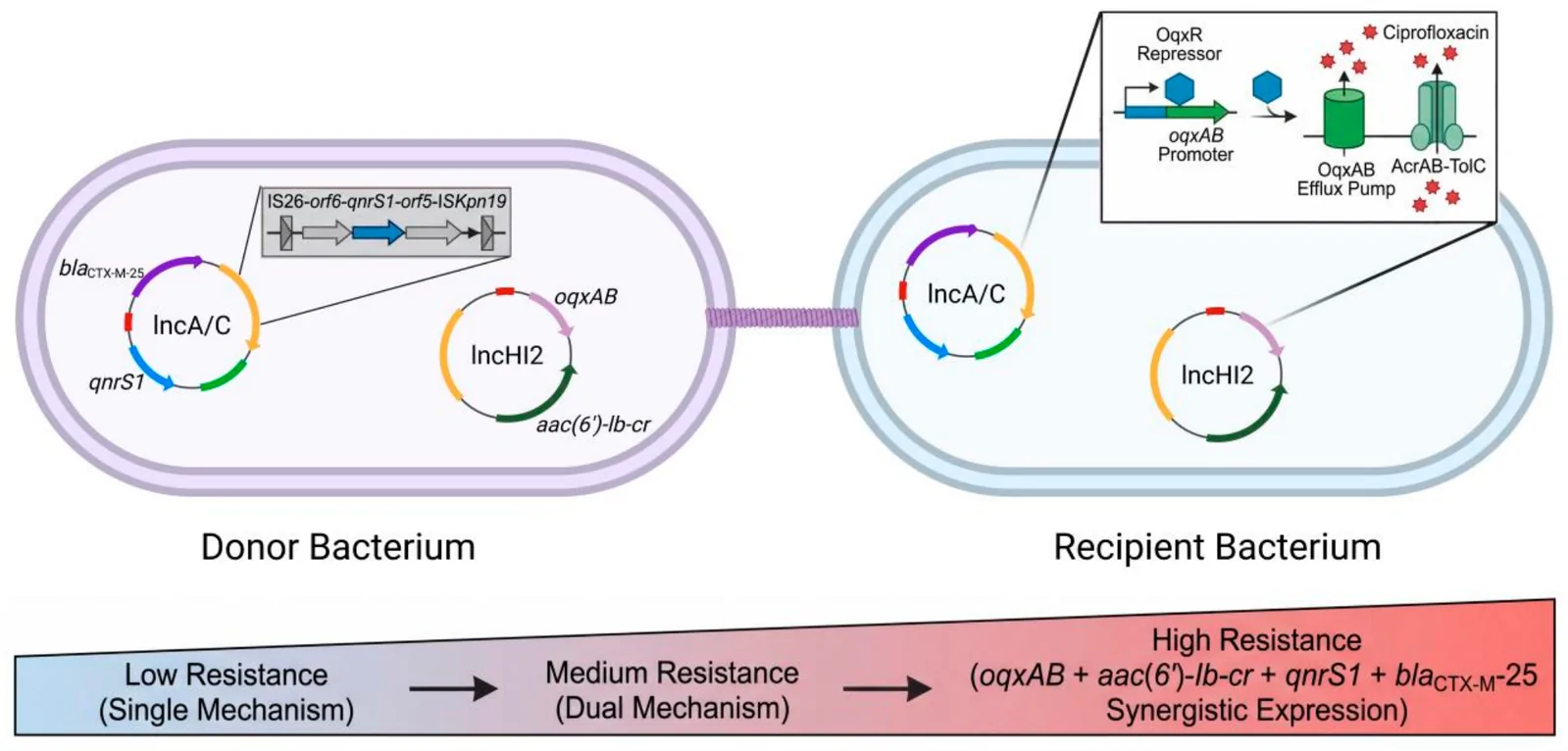
Schematic diagram of plasmid-mediated horizontal transfer of resistance genes and stepwise escalation of drug resistance through synergistic expression of multidrug resistance genes in *Salmonella*. Donor bacteria transfer IncA/C plasmids (harboring *bla*CTX-M-25, *qnrS1*, and IS26-orf6-*qnrS1*-orf5-ISKpn19 composite transposons) and IncHI2 plasmids (harboring *oqxAB* and *aac(6′)-Ib-cr*) to recipient bacteria via conjugation; insufficient repression by OqxR on OqxAB and AcrAB-TolC efflux pumps enhances ciprofloxacin extrusion. The single resistance mechanism confers low-level resistance, and dual mechanisms produce medium-level resistance, whereas synergistic expression of *oqxAB*, *aac(6′)-Ib-cr*, *qnrS1*, and *bla*CTX-M-25 results in high-level multidrug resistance (MDR). *bla*CTX-M-25: extended-spectrum β-lactamase CTX-M-25 gene, *qnrS1*: quinolone resistance protein S1 gene, IS26: insertion sequence 26, IncA/C/IncHI2: incompatibility group A/C/HI2 plasmid, *oqxAB*: multidrug efflux pump OqxAB operon, *aac(6′)-Ib-cr*: aminoglycoside AAC(6′)-Ib-cr 6′-N-acetyltransferase type Ib-cr variant, OqxR: OqxAB repressor, OqxAB AcrAB-TolC: AcrAB-TolC multidrug efflux system, AcrAB-TolC: multidrug resistance. Note: the red symbol, Ciprofloxacin.

Plasmids are small, self-replicating extrachromosomal DNA molecules that propagate resistance genes among bacterial populations via horizontal gene transfer, thereby increasing communal resistance capacities [63]. Plasmid incompatibility (Inc) groups and replicon types govern host range and stability, directly influencing transmission efficiency and plasmid persistence within bacterial communities. In Kentucky, USA, antibiotic resistance genes have been detected on plasmids belonging to the IncI1, IncN, IncFIB, IncFIIA, IncW, IncA/C, and IncL/M incompatibility groups; notably, IncA/C conjugative plasmids carrying the β-lactamase gene *bla*CTX-M-25 have facilitated the regional proliferation of multidrug resistance and high-level fluoroquinolone resistance [64]. The plasmid pSal009, identified in multidrug-resistant *Salmonella* Thompson isolates from ready-to-eat pork products in China, is a transferable IncA/C-type plasmid harboring multiple antibiotic resistance genes, including the fluoroquinolone resistance gene *qnrS1* located within a composite transposon, IS26-orf6-*qnrS1*-orf5-ISKpn19, where orf6 and orf5 encode transposase and recombinase family proteins [60], respectively. The IncHI2 and IncHI2A plasmids represent the predominant lineages driving antibiotic resistance dissemination in *Salmonella*, particularly with respect to fluoroquinolone resistance; IncHI2 plasmids evolve through the acquisition of transposons carrying antibiotic resistance genes, thereby exacerbating resistance propagation [65]. Studies of *Salmonella* Typhimurium isolates from China have demonstrated that *oqxAB* frequently cotransfers with IncHI2 plasmids, enabling the simultaneous transfer of *oqxAB* alongside other resistance genes (e.g., *bla*CTX-M, *qnrS1*) to recipient bacteria and thereby promoting the spread of fluoroquinolone resistance. The transfer of conjugative elements among bacteria enhances the propagation and maintenance of resistance genes within bacterial populations [66]. Plasmid-borne *oqxAB* expression is regulated primarily by the local repressor OqxR; insufficient OqxR expression results in ciprofloxacin-resistant phenotypes in *Salmonella* Typhimurium, and optimal coexpression of *aac(6′)-Ib-cr* and *oqxAB* is required to confer fluoroquinolone resistance [67] (Figure 3).

#### 3.1.2. Genomic Island-Mediated Transfer

Genomic islands are discrete, mobile DNA segments within bacterial genomes that frequently carry multiple antibiotic resistance genes [63]. These islands are typically inserted into chromosomal loci via transposons or insertion sequences (ISs), thereby promoting resistance gene dissemination. *Salmonella* Genomic Island 1 (SGI1) was the first such structure identified, was originally characterized in *Salmonella* Typhimurium DT104, and carries resistance genes conferring resistance to multiple antibiotic classes, including fluoroquinolones. SGI1 can excise from the chromosome via integrase-mediated site-specific recombination to form circular intermediates, which are subsequently transferred to recipient strains through conjugation assisted by helper plasmids [68]. The broad distribution of SGI1 and its variants among diverse *Salmonella* serovars underscores the critical role of genomic islands in resistance gene dissemination.

### 3.2. Transposon-Mediated Horizontal Transfer (Table 1)

#### 3.2.1. Transposon Mobilization

Transposons are mobile DNA elements capable of intragenomic or intergenomic translocation, which is catalyzed by encoded transposases that mediate excision and reintegration at novel genomic positions [63]. Tn7-derived transposons, including Tn6450 and Tn6777, play pivotal roles in resistance propagation. Tn6450 is a newly identified multidrug resistance mobile element in *Salmonella* that carries 18 distinct antibiotic resistance genes, including the fluoroquinolone resistance genes *qnrA1* and *aac(6′)-Ib-cr* [69]. Tn6450 comprises the Tn7 transposition module, a class 2 integron system, and multiple IS26 insertion sequences that facilitate resistance gene accumulation. The presence of a hybrid class 1/2 integron within Tn6450 may promote resistance gene acquisition and transmission. The structural diversity and extensive resistance gene cargo of Tn6450 render it a critical vehicle for resistance dissemination. Additionally, Tn6777, another member of the Tn7 family identified in *Salmonella* Rissen SCSW714, carries multiple antibiotic resistance genes, including fluoroquinolone resistance determinants [70]. The identification of Tn6777 highlights the functional diversity and active mobilization capacity of Tn7-like transposons in resistance propagation. These transposons increase resistance gene expression and dissemination through transposase-mediated chromosomal integration or IS-mediated genetic rearrangement.

#### 3.2.2. Insertion Sequence-Mediated Transfer

Insertion sequences (ISs) represent the simplest transposon type, mobilizing within genomes via transposase activity and causing gene mutation or activation [71]. The key IS elements associated with *Salmonella* resistance dissemination include IS1, IS26, and IS6100. IS1 derivatives likely mediate horizontal gene transfer during the acquisition of the genomic island GI-DT12 [72]. In *Salmonella* Typhimurium DT204 acrB mutants, IS1 or IS10 integration upstream of the acrEF operon resulted in significantly increased expression of the multidrug efflux pump genes *acrEF* [73]. The widely prevalent *oqxAB* genes in *Salmonella* Typhimurium are frequently integrated into plasmids via IS26 transposase-mediated mechanisms [58]. These composite transposons, represented by Tn7 and its derivative structures, leverage transposase activity, integron systems, and multiple IS elements to construct complex resistance transfer platforms in *Salmonella* [70,74]. They not only mediate stable integration and expression of fluoroquinolone resistance genes but also accelerate interstrain resistance dissemination through horizontal gene transfer [75]. Furthermore, IS26 and IS1 increase efflux pump expression and the transmission efficiency of plasmid-borne resistance genes such as *oqxAB* through genetic rearrangement, amplification, and regulatory mechanisms [73]. The continuous discovery of novel transposons such as Tn6450 and Tn6777 underscores the importance of elucidating their mechanistic and evolutionary roles in understanding the emergence and epidemiology of multidrug-resistant phenotypes. The detailed date are summarized in Table 1.

**Table 1:** Overview of mobile genetic elements mediating the horizontal transfer of fluoroquinolone resistance genes in *Salmonella*.

Mobile Genetic Element	Type/Class	Key Structural Features	Role in Fluoroquinolone Resistance Dissemination	Representative Examples	References
Tn6450	Composite transposon (Tn7 family)	Tn7 transposition module + class 2 integron + multiple IS26 copies; hybrid class 1/2 integron	Carries *qnrA1* and *aac(6′)-Ib-cr* within a single mobile platform; promotes resistance gene accumulation and transmission	Identified in *Salmonella* from chicken (China)	[70]
Tn6777	Tn7-like transposon	Tn7-derived backbone with *pco/sil* and resistance gene clusters	Exemplifies structural diversity and active mobilization capacity of Tn7-like elements	*Salmonella* Rissen SCSW714 (porcine isolate)	[71]
IS1	Insertion sequence	Short transposase-encoding element	Mediates HGT during acquisition of genomic island GI-DT12; insertion upstream of *acrEF* activates efflux pump overexpression	*Salmonella* Typhimurium DT204 acrB mutants; GI-DT12 acquisition	[72,73]
IS26	Insertion sequence	Transposase + terminal inverted repeats	Captures *oqxAB* into plasmids (IncF/IncHI2) via transposase-mediated mechanisms; drives genetic rearrangement and amplification	*oqxAB*-positive *Salmonella* Typhimurium; IS26-flanked composite transposons	[55,73]
IS6100	Insertion sequence	/	Contributes to resistance gene mobilization in *Salmonella*	/	/
Class 1 integron	Integron (5′-CS–3′-CS)	*intI1* (integrase), *attI* site, P-cassette promoter, 3′-CS with *qacEΔ1*/*sul1*	IntI1 catalyzes cassette insertion/excision at *attI*/*attC*; fluoroquinolone exposure upregulates IntI1, accelerating QRDR mutation accumulation and cassette capture; frequently harbors *qnrA/B/S*	sul1-type integrons carrying PMQR genes	[76,77,78]

#### 3.2.3. Integron-Mediated Horizontal Transfer

Integrons are mobile genetic elements capable of capturing and expressing exogenous gene cassettes, comprising a 5′-conserved segment (5′-CS) containing an attI recombination site and a 3′-conserved segment (3′-CS) [76]. Class 1 integrons harbor the *intI1* gene, which encodes the integrase IntI1; the expression of IntI1 is responsive to exogenous stressors, including antibiotic exposure, and fluoroquinolone treatment significantly upregulates IntI1 expression, thereby promoting dynamic gene cassette capture and rearrangement and accelerating the adaptive selection of resistance genes. IntI1 specifically recognizes attI sites and attC recombination sites at gene cassette termini, mediating cassette insertion and excision and thereby enabling horizontal transfer of resistance determinants [76,77]. In *Salmonella*, PMQR genes such as *qnrA*/*B*/*S* are frequently incorporated into sul1-type integrons, accompanied by IS1 derivative-mediated recombination on chromosomes or plasmids [77,78]. These characteristics confer genetic advantages for resistance maintenance and enable rapid recombination-mediated acquisition of novel resistance genes under selective pressure.

### 3.3. Antibiotic Selection Pressure

Bacterial populations initially susceptible to antimicrobial agents actively exploit horizontal gene transfer (HGT) to acquire exogenous resistance determinants from adjacent microbial communities, thereby circumventing antibiotic-mediated lethality. Owing to their versatile capacity to acquire and disseminate resistance determinants through multiple genetic avenues, this adaptive mechanism is particularly conspicuous in *Salmonella* [58].

Under antimicrobial selection pressure, diverse mobile genetic elements (MGEs) operate synergistically to orchestrate a highly efficient transmission network for resistance determinants. Insertion sequences (ISs) initially demarcate discrete DNA segments and catalyze homologous recombination, furnishing mobile boundaries and recombination loci for downstream elements [79]. Composite transposons mobilize across replicons via transposase-mediated cut-and-paste or replicative transposition, inserting fluoroquinolone resistance gene cassettes—including *qnr*, *qepA*, *aac(6′)-Ib-cr*, and *oqxAB*—into heterogeneous genomic backbones, thereby affecting interreplicon transfer between chromosomes and plasmids [80,81]. Concurrently, Class 1 integrons exploit IntI1-mediated site-specific recombination at *attI*/*attC* loci to assemble these resistance cassettes into tandem arrays, with transcription driven by the P-cassette promoter. At higher organizational tiers, *Salmonella* Genomic Island 1 (SGI1) captures these integron–transposon chimeras en bloc, integrating them into tRNA loci and subsequently excising them as circular intermediates. Ultimately, conjugative plasmids package transposons, integrons, and genomic island fragments into self-transferable vectors equipped with intact tra/T4SS conjugative transfer modules, thereby facilitating interspecies dissemination and global propagation [82].

**Figure 4:**
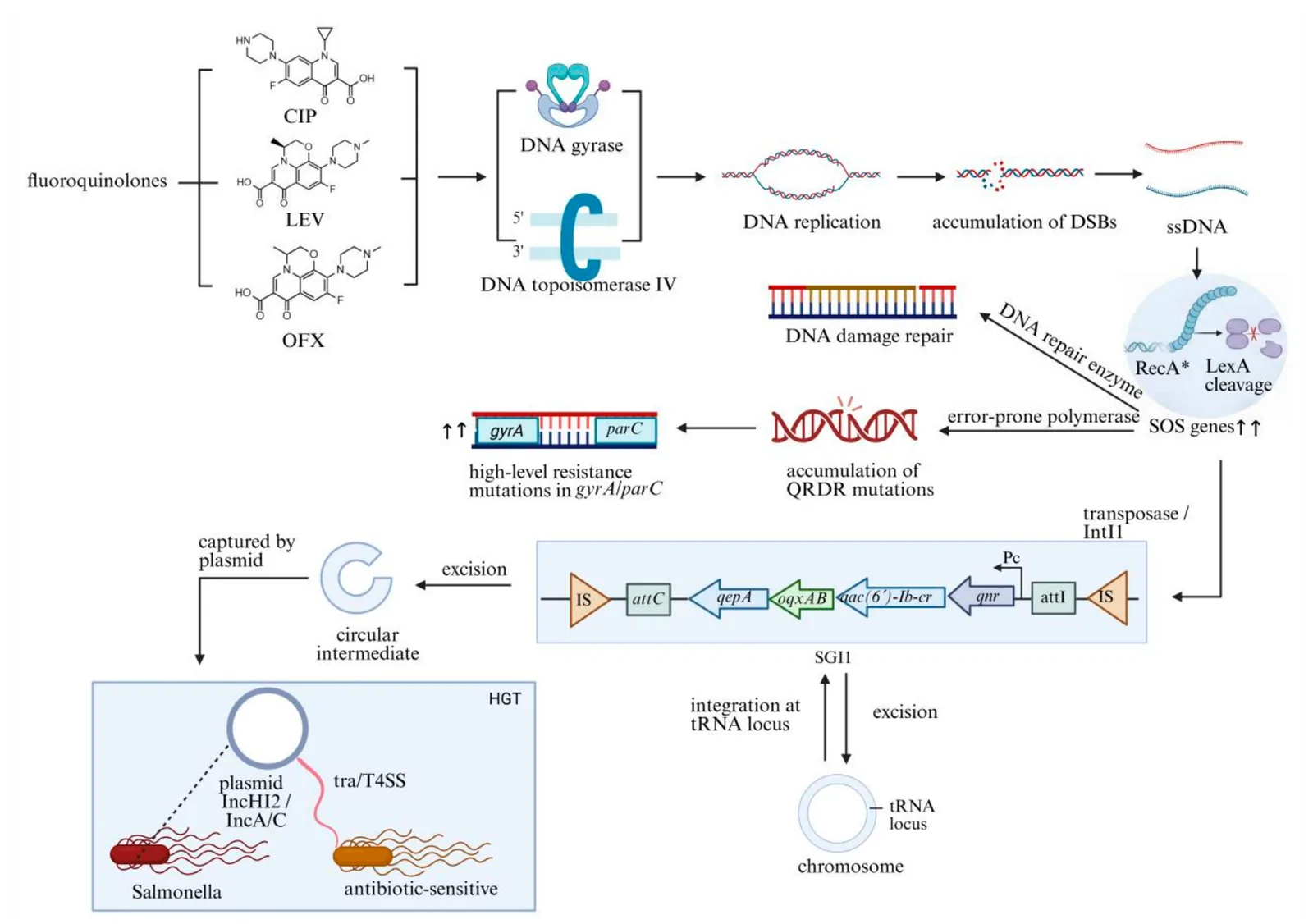
Overview of the fluoroquinolone-induced SOS response and its role in driving the evolution of quinolone resistance in *Salmonella*. Fluoroquinolones (CIP, LEV, OFX) target DNA gyrase and DNA topoisomerase IV, causing DNA double-strand breaks (DSBs) during replication. The accumulation of single-stranded DNA (ssDNA) triggers the formation of RecA* nucleoprotein filaments and LexA autoproteolysis, leading to SOS response activation. The upregulated SOS genes coordinate three parallel pathways: DNA repair enzymes initiate DNA damage repair; error-prone polymerases increase mutation rates, driving the accumulation of quinolone resistance-determining region (QRDR) mutations in gyrA and parC; and transposases/integrases (IntI1) mobilize mobile genetic elements (MGEs). Under antibiotic selection pressure, *Salmonella* Genomic Island 1 (SGI1), a composite transposon flanked by insertion sequences (ISs), integrates into the bacterial chromosome at tRNA loci. Upon excision, SGI1 forms a circular intermediate that is captured by conjugative plasmids (IncHI2/IncA/C) equipped with tra/T4SS modules. The assembled resistance gene cassettes (qnr, qepA, aac(6′)-Ib-cr, oqxAB) within the Class 1 integron platform are subsequently transferred to antibiotic-sensitive recipients via horizontal gene transfer (HGT). Ultimately, fluoroquinolone exposure not only directly selects for chromosomal QRDR mutations but also indirectly accelerates MGE mobilization, enabling *Salmonella* to rapidly acquire and disseminate multidrug resistance under antibiotic pressure. CIP: ciprofloxacin; LEV: levofloxacin; OFX: ofloxacin. RecA*: Activated RecA protein complex. Note: ↑, increase/upregulation. The expression of SOS response-related genes is obviously increased or activated.

Upon fluoroquinolone exposure, the SOS response is activated through RecA nucleoprotein filament formation and LexA autocleavage, which coordinately upregulate the expression of DNA repair enzymes, error-prone polymerases, transposases, and integrases. This dual regulatory axis accelerates both the accumulation of QRDR mutations and the rate of HGT and recombination events, thereby propelling the rapid emergence and dissemination of high-level resistance phenotypes within *Salmonella* populations [83]. This hierarchical, multi-element assembly of ISs, composite transposons, integrons, SGI1, and conjugative plasmids—together with its fluoroquinolone-driven escalation—is illustrated in Figure 4.

## 4. Detection Methodologies

With rapid advancements in molecular biology and analytical technologies, antimicrobial susceptibility assessment has evolved from conventional phenotypic methods to high-throughput, rapid molecular diagnostic methods. Traditional techniques—including disk diffusion, broth microdilution, and agar dilution—remain reliable but are time-consuming and require stringent standardization. To overcome the limitations of conventional methods in terms of speed and sensitivity, novel molecular-based detection technologies have emerged. Matrix-assisted laser desorption/ionization time-of-flight mass spectrometry (MALDI-TOF MS) combined with PCR enables the genotyping of oligonucleotides containing mutation sites through PCR amplification followed by mass spectrometric measurement [84]. Surface-enhanced Raman spectroscopy (SERS) offers high sensitivity, rapid detection, and dynamic monitoring of drug–bacterium interactions, demonstrating substantial potential in microbial identification and resistance analysis [85]. SERS utilizes specific nanostructures as enhancement substrates; when combined with CRISPR technology, Cas protein-responsive nanodevices enable rapid, accurate identification of *Salmonella* Typhimurium and its resistant strains without time-consuming DNA amplification, substantially shortening the detection time. This approach has been validated for detecting reduced fluoroquinolone susceptibility in *Salmonella* Typhi, particularly in strains harboring GyrB mutations [86]. CRISPR/Cas-SERS systems can identify specific resistance-associated gene mutations. Given its simplicity and rapidity, this methodology holds promise for point-of-care testing and global health surveillance, with its high sensitivity and specificity making it an effective tool for combating antimicrobial resistance through global monitoring and research (Table 2).

In summary, the optimal detection method should be selected according to the clinical or surveillance context, infrastructure, and turnaround-time requirements. For resource-limited settings and routine clinical laboratories, disk diffusion and broth microdilution remain, in our assessment, the most appropriate first-line approaches: they require no specialized instrumentation, involve the lowest per-sample costs and operational complexity, and are globally standardized, although their 16–20 h turnaround time constrains rapid decision-making. For tertiary-care centers and reference laboratories, MALDI-TOF MS–based genotyping offers the best balance of throughput, accuracy, and per-sample cost once the capital investment in instrumentation has been made; its complexity is moderate, but instrument purchase and maintenance represent substantial fixed costs that are justifiable only at higher testing volumes. For outbreak investigations and point-of-care screening in endemic regions, CRISPR/Cas-SERS is, in our view, the most promising near-term strategy: it requires no nucleic-acid amplification, returns results within minutes to 2 h, and is compatible with miniaturized, low-cost devices; its current limitations are chiefly operational complexity—dependence on nanostructured enhancement substrates and the need for standardized sample preparation—rather than reagent cost. Flow-cytometric assays occupy an intermediate niche where 2-h results are required, at the expense of high instrument cost. We therefore recommend a tiered algorithm: conventional phenotypic methods as the low-cost baseline for susceptibility testing, MALDI-TOF MS–based approaches for high-throughput reference surveillance, and CRISPR/Cas-SERS for rapid point-of-care detection of reduced fluoroquinolone susceptibility.

## 5. Therapeutic Strategies

The development of novel antibiotics and alternative therapeutics against resistant *Salmonella* represents a critical priority [92]. New antimicrobial agents must demonstrate efficacy against resistant strains while presenting a low propensity for resistance development. Traditional fluoroquinolones—including levofloxacin, ciprofloxacin, and gatifloxacin—were previously first-line therapeutic options for *Salmonella* infections; however, increasing cross-resistance has markedly compromised their clinical utility. Consequently, combination therapeutic strategies leveraging distinct mechanistic synergies represent a promising approach to enhancing antimicrobial efficacy. The cited synergy study used cefotaxime; third-generation cephalosporins should not be treated as interchangeable without direct comparative evidence.

In addition to combination chemotherapy, nonantibiotic therapeutic strategies have considerable potential. Immunotherapy through vaccination reduces infection incidence; probiotics modulate the intestinal microbiota to increase host defenses; and bacteriophage therapy exploits the specific lytic activity of phages, offering novel pathways for treating resistant strains [23] (Table 3).

CRISPR–Cas-based gene editing technologies also present effective avenues for addressing resistance challenges. Research on CRISPR–Cas systems has demonstrated the selective targeting and elimination of antibiotic resistance genes. Furthermore, the exploration of bacteriophages as delivery vehicles for CRISPR–Cas systems may fundamentally transform the therapeutic landscape for bacterial infections [97,98] (Table 4).

In our assessment, these strategies differ substantially in maturity and realistic clinical impact. Among them, optimized combination regimens are the most immediately translatable, as they build on existing drugs and face the lowest regulatory barrier; however, their benefit is constrained by toxicity concerns (e.g., aminoglycoside nephro- and ototoxicity) and the risk of regrowth and further resistance selection. We regard vaccination and probiotic interventions as valuable but fundamentally preventive or adjunctive measures, since neither directly treats established invasive infection. Phage therapy and phage-delivered CRISPR-Cas systems are, scientifically, the most elegant and mechanism-targeted approaches, capable in principle of strain-specific elimination and even plasmid-borne resistance gene removal; nevertheless, in our view they remain the farthest from routine clinical use, pending solutions to delivery efficiency, host-range constraints, off-target effects, and long-term safety within complex microbial ecosystems. We therefore caution against presenting any single non-antibiotic modality as a replacement for antibiotics at present; rather, the realistic near-term goal is an integrated strategy in which rapid diagnostics, stewardship-guided combination therapy, and adjunctive phage or CRISPR-based interventions are deployed in a coordinated manner.

## 6. Prevention and Control

To effectively curtail resistance dissemination, rigorous antimicrobial stewardship programs (ASPs) must be implemented across all sectors, prioritizing narrow-spectrum, targeted antibiotics to reduce empirical and nontargeted prescribing. Antibiotic usage—particularly agents directly relevant to human infection treatment—should be minimized in human medicine, veterinary medicine, and agriculture [90]. Healthcare facilities must enforce strict hand hygiene protocols, environmental cleaning and disinfection, and appropriate isolation measures to reduce nosocomial transmission of resistant organisms. Public education regarding rational antibiotic use, personal hygiene, and food safety can reduce community transmission risks [101]. Surveillance and research on resistance evolution constitute critical components of prevention and control. Establishing and refining antimicrobial resistance surveillance networks; constructing high-throughput resistance monitoring platforms spanning human, animal, and environmental domains; and integrating whole-genome sequencing with epidemiological data enable timely detection of resistance trends, thereby providing scientific evidence for the formulation of prevention and control strategies [102].

## 7. Challenges and Future Perspectives

The widespread clinical utilization of fluoroquinolones has driven a continuous increase in *Salmonella* resistance. High-level resistance to first-line ciprofloxacin in fluoroquinolone-resistant *Salmonella* primarily results from the synergistic accumulation of chromosomal *gyrA*/*parC* mutations and plasmid-mediated PMQR genes (e.g., *qnr*, *aac(6′)-Ib-cr*), leading to the therapeutic failure of ciprofloxacin and an escalating threat from fluoroquinolone-resistant *Salmonella* [103]. This underscores the urgent need for global action to preserve the efficacy of existing antibiotics and develop novel therapeutic strategies to ensure the sustained treatment of life-threatening infections [104].

The complexity of resistance mechanisms—including target site alterations, efflux pump overexpression, and plasmid-mediated resistance gene dissemination—renders therapeutic intervention increasingly difficult. Moreover, the interbacterial transmission of resistance genes via conjugative plasmids, transposons, and other mobile genetic elements substantially complicates resistance control [105]. Although in vitro studies have achieved selective clearance of resistance plasmids and targeted gene editing, critical challenges remain regarding vector delivery efficiency, off-target effects, and long-term safety assessment within complex microbial ecosystems. Future research should focus on the rational design of novel efflux pump inhibitors, the optimization of artificial intelligence-assisted resistance prediction models, and the establishment of multicenter clinical trial frameworks, with the ultimate goal of achieving precise prevention, control, and therapeutic innovation against fluoroquinolone-resistant *Salmonella*.

From our perspective, each resistance mechanism identified in this review admits at least one corresponding countermeasure, although these differ markedly in maturity. For chromosomal QRDR mutations in *gyrA/parC*—which cannot be directly reversed—the practical countermeasures are stewardship-guided substitution of non-quinolone agents (third-generation cephalosporins, azithromycin) and typhoid conjugate vaccination to prevent infection at the population level; both are already in clinical use and have demonstrated benefit in regions affected by extensively drug-resistant typhoid. For efflux pump overexpression (AcrAB-TolC, OqxAB), efflux pump inhibitors such as PAβN—already validated experimentally to restore ciprofloxacin susceptibility—represent the most direct countermeasure, and clinical candidates are under development. For enzymatic modification by AAC(6′)-Ib-cr, CRISPR-Cas systems targeting *aac(6′)-Ib-cr*–harboring plasmids offer, in principle, a specific and self-amplifying solution, though delivery remains the principal obstacle. For reduced outer-membrane permeability, permeabilizing adjuvants that restore drug access to the periplasm are a rational, if still preclinical, countermeasure. For PMQR determinants carried on conjugative plasmids, plasmid-curing compounds and phage-delivered CRISPR are the most mechanistically elegant countermeasures, with strong in vitro proof-of-concept. Finally, for MGE-driven horizontal transfer itself, SOS-response inhibitors (targeting the RecA–LexA axis) and anti-conjugation strategies aim to cut the transmission route rather than the resistance gene. Collectively, these strategies fall into three tiers: those already in use and effective (stewardship, vaccination, alternative antibiotic therapy); promising near-term candidates (efflux pump inhibitors, optimized combination regimens, rapid CRISPR-based diagnostics); and longer-term, high-reward approaches (phage-delivered CRISPR, plasmid curing, SOS inhibition).

## 8. Conclusions

The global dissemination of fluoroquinolone-resistant *Salmonella* constitutes a severe threat to public health. Reduced fluoroquinolone susceptibility and the emergence of high-level resistance are associated with chromosomal mutations in DNA gyrase and topoisomerase genes (particularly *gyrA* and *parC*), overexpression of multidrug efflux pumps, plasmid-mediated resistance mechanisms, and horizontal interbacterial transmission via plasmids, transposons, and other mobile genetic elements. Global coordinated action, strengthened public health policies, implementation of surveillance and control measures, and enhanced international collaboration are essential for preserving existing antibiotic efficacy, developing novel therapeutic strategies, mitigating the transmission of resistant clones, and ultimately safeguarding public health security. Correspondingly, countermeasures fall into three tiers: measures already in clinical use (antimicrobial stewardship, typhoid conjugate vaccination, and alternative antibiotic therapy), promising near-term candidates (efflux-pump inhibitors, optimized combination regimens, and rapid molecular diagnostics), and longer-term, high-reward approaches (phage-delivered CRISPR-Cas, plasmid curing, and SOS-response inhibition). The development of new antibiotics represents not only a scientific challenge but also a societal and ethical imperative requiring concerted efforts from the global scientific community and public health organizations.

## Figures and Tables

**Table 2:** Comparison of phenotypic antimicrobial susceptibility testing and rapid molecular/spectroscopic platforms for detecting fluoroquinolone resistance in *Salmonella*.

Method	Subtype/Platform	Time	LOD (CFU/mL)	Amplification Required	Advantages	References
Disk diffusion (Kirby-Bauer)		16–18 h	—(MIC/zone diameter/S-I-R endpoint)	No	Most economical and easy to operate; globally standardized	[87]
Broth microdilution		20 h	—(MIC/zone diameter/S-I-R endpoint)	No	MIC quantification; capable of parallel testing of multiple antibiotics; good reproducibility	[88]
Agar dilution		16–20 h	—(MIC/zone diameter/S-I-R endpoint)	No	MIC quantification; suitable for screening large batches of strains	[89]
Flow cytometric fluorescence assay		2 h	1–10	No	Rapid; high sensitivity; detectable at low bacterial concentrations	[23,90]
MALDI-TOF MS		1–6 h	10^3^–10^6^	Yes	Multilocus high-throughput; high accuracy	[84]
SERS	Nanointegrated CRISPR/Cas-SERS	<2 h	10^2^	No	High specificity; rapid	[85,86]
SERS	Fiber-optic integrated SERS	<10 min	10^−1^	No	Miniaturized and portable; extremely high sensitivity	[91]

**Table 3:** Progress in Combination Therapies against Fluoroquinolone-Resistant *Salmonella* (CLSI breakpoints).

Combination Regimen	Drug A Breakpoint (CLSI, S/I/R, μg/mL)	Drug B Breakpoint (CLSI, S/I/R, μg/mL)	Synergy Evaluation	Major Limitations	References
Ciprofloxacin + Cefotaxime	≤0.06/>0.06	≤1/I = 2/>2	Not reported	Requires high-dose administration to maintain synergistic concentrations; some strains exhibit regrowth after 48–72 h; clinical treatment requires monitoring of drug concentrations and toxicity	[93]
Ciprofloxacin + Gentamicin	≤0.06/>0.06	≤2/—/>2	FIC index range 0.121–0.216 (≤0.5), showing high synergy	High risk of ototoxicity and nephrotoxicity with gentamicin (AKI incidence up to 10–25%); strict monitoring of serum drug concentrations and renal function required	[94]
Meropenem + Fosfomycin	≤2/>8	≤32/—/>32	FIC < 0.5, significant synergy	Prone to induce resistance	[95]
Marbofloxacin + Methyl gallate	/	CLSI VET01	0.5	Still at the in vitro or animal model research stage; no clinical experience available; further studies on pharmacokinetics and safety required	[96]

**Table 4:** Clinical and experimental strategies against fluoroquinolone-resistant *Salmonella*.

Strategy	Target/Pathway	Evidence Maturity	Key Limitations	Representative References
Stewardship-guided non-quinolone therapy (e.g., third-generation cephalosporins, azithromycin)	Replacement of fluoroquinolones for invasive typhoidal salmonellosis; reduces selective pressure	In clinical use; recommended for drug-resistant/XDR typhoid fever	Requires rapid diagnostics and local susceptibility data; limited options for XDR strains	[14,38,90]
Optimized combination regimens (e.g., ciprofloxacin + cefotaxime; meropenem + fosfomycin)	Dual targeting to enhance bacterial killing and suppress regrowth	Limited; in vitro synergy studies and individual case reports	Toxicity (e.g., aminoglycoside nephro-/ototoxicity); regrowth and resistance selection after 48–72 h; therapeutic drug monitoring required	[93,94,99,100]
Typhoid conjugate vaccination	Prevention of typhoidal *Salmonella* infection at the population level	Established preventive intervention	Does not treat established invasive infection; coverage restricted to typhoidal serovars	[99]
Probiotics/microbiota modulation	Restoration of gut barrier function and competitive exclusion of pathogens	Adjunctive; clinical evidence weak	Does not directly clear invasive *Salmonella*; strain-specific effects	[3]
Bacteriophage therapy	Strain-specific lytic killing of *Salmonella*	Experimental; in vitro/animal studies and limited reports	Host-range constraints; receptor escape; formulation, purification, and regulatory hurdles	[23,100]
Phage-delivered CRISPR-Cas/plasmid curing	Targeted removal of PMQR-bearing plasmids or resistance genes	In vitro proof-of-concept	Delivery efficiency; off-target effects; long-term safety within complex microbial ecosystems	[92,97,98]
Efflux-pump inhibition (e.g., PAβN-class inhibitors)	Blockade of AcrAB-TolC/OqxAB-mediated fluoroquinolone extrusion	Preclinical; restores ciprofloxacin susceptibility in vitro	Toxicity and selectivity; pharmacokinetic optimization required	[21,25,59]
Anti-SOS/anti-conjugation adjuvants; outer-membrane permeabilizers	Suppression of QRDR mutation accumulation and horizontal transfer; restoration of periplasmic drug access	Preclinical	Narrow activity windows; potential for adjuvant resistance; limited in vivo validation	[33,54,82,83]

## Data Availability

No new data were created or analyzed in this study. Data sharing is not applicable to this article.
